# Antimicrobial susceptibility and molecular epidemiology of clinical *Enterobacter cloacae* bloodstream isolates in Shanghai, China

**DOI:** 10.1371/journal.pone.0189713

**Published:** 2017-12-15

**Authors:** Su Wang, Shu-Zhen Xiao, Fei-Fei Gu, Jin Tang, Xiao-Kui Guo, Yu-Xing Ni, Jie-Ming Qu, Li-Zhong Han

**Affiliations:** 1 Department of Clinical Microbiology, Ruijin Hospital, Shanghai Jiao Tong University School of Medicine, Shanghai, China; 2 Department of Clinical Laboratory, Shanghai Jiao Tong University Affiliated Sixth People’s Hospital, Shanghai, China; 3 Department of Medical Microbiology and Parasitology, Shanghai Jiao Tong University School of Medicine, Shanghai, China; 4 Department of Pulmonary Medicine, Ruijin Hospital, Shanghai Jiao Tong University School of Medicine, Shanghai, China; Seconda Universita degli Studi di Napoli, ITALY

## Abstract

**Background:**

*Enterobacter cloacae* is a major nosocomial pathogen causing bloodstream infections. We retrospectively conducted a study to assess antimicrobial susceptibility and phylogenetic relationships of *E*. *cloacae* bloodstream isolates in two tertiary university-affiliated hospitals in Shanghai, in order to facilitate managements of *E*. *cloacae* bloodstream infections and highlight some unknowns for future prevention.

**Methods:**

Fifty-three non-duplicate *E*. *cloacae* bloodstream isolates were consecutively collected from 2013 to 2016. Antimicrobial susceptibility was determined by disk diffusion. PCR was performed to detect extended-spectrum β-lactamase (ESBL), carbapenemase and colistin resistance (MCR-1) gene. Plasmid-mediated AmpC β-lactamase (pAmpC) genes were detected using a multiplex PCR assay targeting MIR/ACT gene (closely related to chromosomal EBC family gene) and other plasmid-mediated genes, including DHA, MOX, CMY, ACC, and FOX. eBURST was applied to analyze multi-locus sequence typing (MLST).

**Results:**

The rates of resistance to all tested antibiotics were <40%. Among 53 *E*. *cloacae* isolates, 8(15.1%) were ESBL producers, 3(5.7%) were carbapenemase producers and 18(34.0%) were pAmpC producers. ESBL producers bear significantly higher resistance to cefotaxime (100.0%), ceftazidime (100.0%), aztreonam (100.0%), piperacillin (87.5%), tetracycline (75.0%), and trimethoprim-sulfamethoxazole (62.5%) than non-producers (*p*<0.05). PAmpC- and non-producers both presented low resistance rates (<40%) to all antibiotics (p>0.05). SHV (6/8, 75.0%) and MIR/ACT (15/18, 83.3%) predominated in ESBL and pAmpC producers respectively. Moreover, 2 isolates co-carried TEM-1, SHV-12, IMP-26 and DHA-1. MLST analysis distinguished the 53 isolates into 51 STs and only ST414 and ST520 were assigned two isolates of each (2/53).

**Conclusion:**

The antimicrobial resistance rates were low among 53 *E*. *cloacae* bloodstream isolates in the two hospitals. Multiclonality disclosed no evidence on spread of these isolates in Shanghai. The simultaneous presence of ESBL, carbapenemase and pAmpC detected in 2 isolates was firstly reported in Shanghai, which necessitated active ongoing surveillances and consistent prevention and control of *E*. *cloacae*.

## Introduction

*Enterobacter cloacae* is an important emerging pathogen, causing various nosocomial infections, including respiratory infections, bloodstream infections (BSIs) and surgical site infections [1, 2]. BSIs due to multidrug-resistant (MDR) *Enterobacteriaceae* were related to high mortality, sometimes exceeding 50% depending on the study population[3]. *E*. *cloacae* has become the third most frequent and lethal *Enterobacteriaceae* species involved in BSIs over the past decades[4–6]. Worse than that, MDR *E*. *cloacae* isolates have been reported widespread, posing a serious threat to empiric therapy[6–9]. An assessment showed global resistance to cefepime increased significantly from 36% in 2004 to 63% in 2014 and 8.5% of *E*. *cloacae* were MDR in Asia-Pacific Rim, just lower than that in Latin America (14%) [10].

The factors dominantly contributing to resistance among *E*. *cloacae* maybe the plasmid-mediated AmpC β-lactamases (pAmpC), plasmid-encoded CTX-M family of extended-spectrum β-lactamases(ESBLs), the KPC family of carbapenemases, and metallo β-lactamases of the VIM, IMP, and NDM-1 types[6, 11]. Production of ESBLs and pAmpC by *E*. *cloacae* were continuously documented worldwide [12]. For example, 42% *E*. *cloacae* bloodstream isolates were screened as ESBL positive in Brazil with CTX-M-15 the most common type [13]. In Australia, IMP-producing *E*. *cloacae* has been noted to be the predominant form of carbapenemase-producing *Enterobacteriaceae* (CPE) [14].

These sporadic cases of previously discovered enzymes and new reports of resistant genes necessitated resistance surveillance and molecular characterization on *E*. *cloacae* to identify potentially endemic resistance genes before their spread. Nevertheless, scarce data on susceptibility and molecular epidemiology of *E*. *cloacae* causing BSIs were available in Shanghai, even in China. This study, therefore, is to give a broad scope on the antimicrobial susceptibility and the presence of resistance genes and their relationships with sequence types (STs) among local *E*. *cloacae* bloodstream isolates. It was the first study on phenotypic and molecular properties of these isolates in Shanghai.

## Materials and methods

### Setting and isolates

This study was conducted in two comprehensive tertiary university-affiliated hospitals (Hospital A and B). They served a local population of around 1.8 million, located in Huangpu District (Hospital A, 1800 beds) and Xuhui District (Hospital B, 1950 beds) respectively. Fifty-three consecutive and non-duplicate *E*. *cloacae* isolates were collected from January 2013 to June 2016 (34 from Hospital A and 19 from Hospital B). All isolates were recovered from blood and re-identified using Matrix Assisted Laser Desorption Ionization-Time of Flight mass spectrometry (MALDI-TOF MS) (bioMérieux, Mar-cyl’Étoile, France) combining with classical biochemical tube tests (Melibiose fermentation test)[15].

The study was approved by Ruijin Hospital Ethics Committee (Shanghai Jiao Tong University School of Medicine), and the Review Board exempted request for informed consent because our study only focused on the bacteria and no patient-level data were involved.

### Antimicrobial susceptibility testing

Antimicrobial susceptibility of the *E*. *cloacae* isolates were determined by the disk diffusion as recommended by CLSI [16]. The antibiotics tested were piperacillin, piperacillin-tazobactam, cefepime, cefotaxime, ceftazidime, aztreonam, imipenem, meropenem, ertapenem, amikacin, gentamicin, sulfamethoxazole-trimethoprim, ciprofloxacin, levofloxacin, tetracycline, tigecycline and polymyxin B. Results were interpreted according to CLSI criteria (M100S 26^th^ Edition)[16]. For tigecycline, the result was interpreted using the European Committee On Antimicrobial Susceptibility Testing (EUCAST) criteria (2017 Ver. 7.0)[17]; for polymyxin B, the result was interpreted using the Antibiogram Committee of the French Society for Microbiology criteria (CASFM 2013 Ver. June [http://www.sfm-microbiologie.org/UserFiles/files/casfm/CASFM2013vjuin.pdf]). *Escherichia coli* ATCC 25922 was used for quality control.

### Detection of resistance genes

Polymerase chain reaction (PCR) was performed to confirm the existence of ESBL genes (*bla*_TEM_, *bla*_SHV_, *bla*_CTX-M (-1,-9 group)_, *bla*_OXA (-1,-2,-10 group)_, *bla*_VEB_, *bla*_GES_, and *bla*_PER_), carbapenemase genes (*bla*_VIM_, *bla*_IMP_, *bla*_KPC_, *bla*_OXA-48_, and *bla*_NDM_) and plasmid-mediated colistin resistance gene (*mcr-1*), using primers previously described [18, 19]. The presence of pAmpC genes was detected using a multiplex PCR assay targeting MIR/ACT (closely related with chromosomal EBC family gene) and other plasmid-mediated genes, including DHA, MOX, CIT, ACC and FOX, as described by Pérez-Pérez and Hanson[20]. Positive amplicons were bidirectional sequenced and aligned with subtypes of resistance genes in GenBank (http://blast.ncbi.nlm.nih.gov/BLAST).

### MLST

A multilocus sequence typing (MLST) scheme was used to assign *E*. *cloacae* isolates to clonal lineages, including seven conserved housekeeping genes (*dnaA*, *fusA*, *gyrB*, *leuS*, *pyrG*, *rplB*, and *rpoB*) as described by Miyoshi-Akiyama et al.[21]. The combination of seven alleles can define the ST for one isolate according to the MLST website (http://pubmlst.org/ecloacae/). STs not found in the database were submitted. The eBURST version 3.0 was used to analyze the clustering of related STs. The eBURST algorithm can group strains according to their allelic profiles by employing a user-specified group definition, as well as drawing a rough sketch to show the genetic relationship[22]. In this study, if 6 of the 7 alleles were homologous, strains would be grouped together.

### Statistical analysis

SAS 8.2 (SAS Institute Inc., Cary, NC, USA) was used for statistical analysis. Continuous variables were presented as median and interquartile range. The chi-square or Fisher’s exact test was used to compare the disparity between different groups for categorical variables. Differences were considered statistically significant at a two-tailed *P* value of <0.05.

## Results

### Clinical data

The age of the 53 patients ranged from 23 to 86 years (median age: 59.4 years, interquartile range: 50–70 years). Male patients (37/53) were more than females (16/53). Near half of the episodes were derived from the surgery (22/53), and where most patients suffered from malignant tumor (10/22) (S1 Table).

### Antimicrobial susceptibility testing

As summarized in Table 1, the rates of resistance to all tested antibiotics were <40% and cefotaxime exhibited the highest resistance (37.7%). Among 53 *E*. *cloacae* isolates, 8(15.1%) were ESBL producers, 3(5.7%) were carbapenemase producers and 18(34.0%) were pAmpC producers (Table 2). The highest resistance rates of ESBL producers were to cefotaxime (100.0%), ceftazidime (100.0%), aztreonam (100.0%), piperacillin (87.5%), tetracycline (75.0%), and trimethoprim-sulfamethoxazole (62.5%). Simultaneously, ESBL producers bear significantly higher resistance to them than non-producers (*P*<0.05). Of note, pAmpC- and non-producers both presented low resistance rates (<40%) to all antibiotics, which did not differ significantly in the two groups (p>0.05) (Table 1).

**Table 1:** Rates of antimicrobial resistance in 53 *E*. *cloacae* bloodstream isolates.

Antibiotics	Number of isolates (%)			*P*	Number of isolates (%)			*P*
	Total (N = 53)	ESBL (N = 8)	non-ESBL (N = 45)		Total (N = 53)	pAmpC (N = 18)	non-pAmpC (N = 35)	
piperacillin	16 (30.2)	7 (87.5)	9 (20.0)	0.0006	16 (30.2)	5 (27.8)	11 (31.4)	0.7839
piperacillin-tazobactam	6 (11.3)	2 (25.0)	4 (8.9)	0.2195	6 (11.3)	2 (11.1)	4 (11.4)	1.0000
aztreonam	18 (34.0)	8 (100.0)	10 (22.2)	0.0001	18 (34.0)	6 (33.3)	12 (34.3)	0.9447
cefepime	7 (13.2)	4 (50.0)	3 (6.7)	0.0056	7 (13.2)	4 (22.2)	3 (8.6)	0.3362
imipenem	1 (1.9)	1 (12.5)	0 (0.0)	0.1509	1 (1.9)	0 (0.0)	1 (2.9)	1.0000
meropenem	3 (5.7)	3 (37.5)	0 (0.0)	0.0024	3 (5.7)	2 (11.1)	1 (2.9)	0.5459
ertapenem	3 (5.7)	3 (37.5)	0 (0.0)	0.0024	3 (5.7)	2 (11.1)	1 (2.9)	0.5459
tigecycline	2 (3.8)	0 (0.0)	2 (4.4)	1.0000	2 (3.8)	0 (0.0)	2 (5.7)	0.5428
gentamicin	5 (9.4)	4 (50.0)	1 (2.2)	0.0011	5 (9.4)	4 (22.2)	1 (2.9)	0.0738
amikacin	2 (3.8)	2 (25.0)	0 (0.0)	0.0203	2 (3.8)	2 (11.1)	0 (0.0)	0.1110
tetracycline	8 (15.1)	6 (75.0)	2 (4.4)	< .0001	8 (15.1)	5 (27.8)	3 (8.6)	0.1486
ciprofloxacin	2 (3.8)	2 (25.0)	0 (0.0)	0.0203	2 (3.8)	1 (5.6)	1 (2.9)	1.0000
levofloxacin	2 (3.8)	2 (25.0)	0 (0.0)	0.0203	2 (3.8)	1 (5.6)	1 (2.9)	1.0000
sulfamethoxazole-trimethoprim	10 (18.9)	5 (62.5)	5 (11.1)	0.0034	10 (18.9)	6 (33.3)	4 (11.4)	0.1189
ceftazidime	16 (30.2)	8 (100.0)	8 (17.8)	< .0001	16 (30.2)	6 (33.3)	10 (28.6)	0.7206
cefotaxime	20 (37.7)	8 (100.0)	12 (26.7)	0.0004	20 (37.7)	7 (38.9)	13 (37.1)	0.9012
polymyxin B	8 (11.9)	1 (12.5)	4 (8.9)	0.5743	8 (11.9)	2 (11.1)	3 (8.6)	1.0000

**Table 2:** Resistance genes in *E*. *cloacae* isolates from bloodstream infections within Two Hospitals.

Types of resistance genes			Numbers of isolates (%)		
			Total (N = 53)	HA (N = 34)	HB (N = 19)
**ESBL**			**8 (15.1)**	**7 (20.6)**	**1 (5.3)**
	SHV	SHV-12	6 (75.0)	5 (71.4)	1 (100.0)
	CTX		2 (25.0)	2 (28.6)	0 (0.0)
		CTX-15	1 (50.0)	1 (50.0)	0 (-)
		CTX-65	1 (50.0)	1 (50.0)	0 (-)
**Carbapenemase**			**3 (5.7)**	**3 (8.8)**	**0 (0.0)**
	IMP	IMP-26	2 (66.7)	2 (66.7)	0 (-)
	NDM	NDM-1	1 (33.3)	1 (33.3)	0 (-)
**AmpC**			**18 (34.0)**	**12 (35.3)**	**6 (31.6)**
ACT/MIR			15 (83.3)	10 (83.3)	5 (83.3)
	ACT		12 (80.0)	7 (70.0)	5 (100.0)
		ACT-20	5 (41.7)	4 (57.1)	1 (20.0)
		ACT-3	3 (25.0)	2 (28.6)	1 (20.0)
		ACT-13	1 (8.3)	0 (0.0)	1 (20.0)
		ACT-8	1 (8.3)	0 (0.0)	1 (20.0)
		ACT-21	1 (8.3)	0 (0.0)	1 (20.0)
		ACT-38	1 (8.3)	1 (14.3)	0 (0.0)
	MIR		3 (20.0)	3 (30.0)	0 (0.0)
DHA	DHA	DHA-1	3 (16.7)	2 (16.7)	1 (16.7)
**Others**	TEM	TEM-1	**5 (9.4)**	**5 (14.7)**	**0 (0.0)**

HA, Hospital A; HB, Hospital B.

### Resistance genes

Of the 8 ESBL producers, SHV enzymes (6, 75.0%) predominated with all identified as SHV-12, and CTX-M enzymes (2, 25.0%) followed, including CTX-M-15(1, 50.0%) and CTX-M-65 (1, 50.0%) (Table 2). A total of 18 (34.0%) of 53 *E*. *cloacae* isolates were positive for pAmpC genes, of which 15 (83.3%) isolates were detected with MIR/ACT gene and the other 3 (16.7%) were detected with DHA-1 gene (Table 2). Of the 15 MIR/ACT producers, the dominant was ACT (12, 80.0%), and MIR (3, 20.0%) followed. The most common subtypes of ACT were ACT-20 (5, 41.7%) and ACT-3 (3, 25.0%). There were also 3 carbapenemase producers (1 NDM-1 and 2 IMP-26 producers) co-carrying SHV-12 (Table 3). Besides, 5 TEM-1-producing isolates were also identified. No *bla*_OXA (-1,-2,-10 group)_, *bla*_VEB_, *bla*_GES_, *bla*_PER_, *bla*_VIM_, *bla*_KPC_, *bla*_OXA-48_ and *bla*_mcr-1_ were detected.

**Table 3:** Antimicrobial resistance profiles and genotypes in MLST of 53 *E*. *cloacae* isolates from bloodstream infections.

ST	Total number	Hospital	Resistance genes					Antimicrobial resistance profiles
			ESBL	Carbapenemase	EBC	DHA	Others	
ST414	2	B	SHV-12					PRL-ATM-GM-TE-CAZ-CTX
		A					TEM-1	PRL-FEP-CTX
ST520	2	A						-
ST22	1	B			ACT-3			-
ST32	1	B						ATM-CAZ-CTX-PB
ST41	1	A			MIR-1			-
ST45	1	A						-
ST46	1	B						TGC
ST62	1	B						PRL-ATM-SXT-CTX
ST65	1	B			ACT-20			-
ST78	1	A						-
ST84	1	A						-
ST88	1	B						-
ST113	1	A			ACT-20			TZP-GM-TE-SXT
ST114	1	B						-
ST118	1	B			ACT-21			-
ST133	1	A	SHV-12		ACT-20			PRL-ATM-TE-SXT-CAZ-CTX
ST175	1	A						PRL-SXT-CTX
ST177	1	A	CTX-65		ACT-20		TEM-1	PRL-ATM-FEP-GM-AK-TE-LEV-SXT-CAZ-CTX
ST191	1	A	SHV-12	IMP-26		DHA-1	TEM-1	PRL-ATM-FEP-MEM-ETP-GM-TE-SXT-CAZ-CTX
ST254	1	A						PRL-TZP-ATM-CAZ-CTX
ST279	1	A	CTX-15					PRL-TZP-ATM-CAZ-CTX-PB
ST318	1	A						-
ST365	1	A						-
ST418	1	A	SHV-12	NDM-1			TEM-1	PRL-TZP-ATM-FEP-IPM-MEM-ETP-TE-CIP-LEV-SXT-CAZ-CTX
ST422	1	A			MIR-1			-
ST524	1	A						PB
ST528	1	A	SHV-12	IMP-26		DHA-1	TEM-1	PRL-ATM-FEP-MEM-ETP-GM-AK-TE-SXT-CAZ-CTX
ST533	1	B						-
ST536	1	A						PRL-ATM-CAZ-CTX
ST562	1	A			ACT-3			-
ST584	1	B						-
ST636	1	B						ATM
ST691	1	B						-
ST718	1	B						-
ST744	1	A	SHV-12					ATM-CAZ-CTX
ST777	1	A						-
ST852	1	A						PRL-ATM-TE-SXT-CAZ-CTX
ST853	1	A						-
ST854	1	A						-
ST855	1	A						-
ST856	1	A			ACT-20			PRL-TZP-ATM-FEP-CAZ-CTX
ST860	1	B			ACT-13			-
ST861	1	B			ACT-8			-
ST862	1	B						PRL-ATM-TGC-CAZ-CTX
ST863	1	B				DHA-1		-
ST864	1	B						-
ST865	1	A			ACT-38			ATM-CAZ-CTX
ST866	1	A						PRL-TZP-ATM-FEP-CAZ-CTX
ST870	1	A			MIR-1			PB
ST871	1	A			ACT-3			SXT-CTX-PB
ST872	1	A						-

PRL, piperacillin; TZP, piperacillin-tazobactam; ATM, aztreonam; FEP, cefepime; IPM, imipenem; MEM, meropenem; ETP, ertapenem; TGC, tigecycline; GM, gentamicin; AK, amikacin; TE, tetracycline; CIP, ciprofloxacin; LEV, levofloxacin; SXT, trimethoprim-sulfamethoxazole; CAZ, ceftazidime; CTX, cefotaxime; PB, polymyxin B.

### MLST

**Fig 1:**
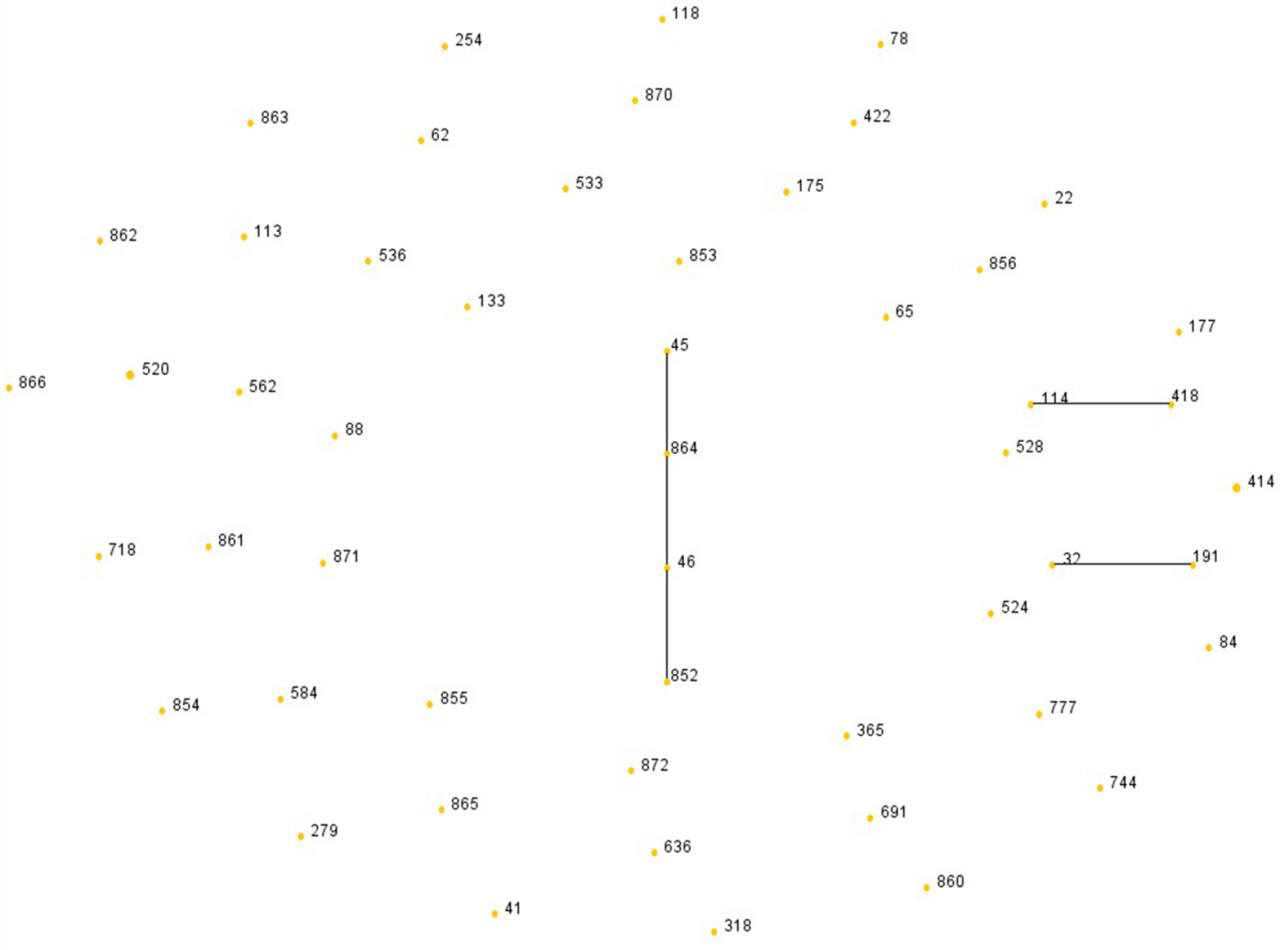
The rough sketch produced by eBURST with the stringent (default) group definition, representing 53 *E*. *cloacae* bloodstream isolates: there were 43 singletons, 3 CCs(CC1: ST45, ST46, ST852, ST864; CC2: ST32, ST191; CC3: ST114, ST418). The line distances had no significance and the area of each yellow circle corresponded to the prevalence of the STs in the MLST data of this study.

MLST analysis distinguished 51 different STs, clustered into 3 non-overlapping clonal complexes (CCs) and 43 singletons (Fig 1). Only ST414 (2/53) and ST520 (2/53) were isolated two of each ST, while the others were assigned to an isolate per ST (Table 3). Fifteen STs (ST852 to 856, ST860 to 866, ST870 to 872) were newly identified whose sequences have been submitted to the database (https://pubmlst.org/bigsdb?db=pubmlst_ecloacae_seqdef). It was indicated in Table 3 that 5 isolates harbored two or more types of enzymes, including ST133 (SHV-12 and ACT-20), ST418 (TEM-1, SHV-12 and NDM-1), ST177 (CTX-M-65 and ACT-20), ST191 and ST528 (TEM-1, SHV-12, IMP-26 and DHA-1).

## Discussion

*E*. *cloacae* was frequently implicated in nosocomial infections[1, 2], and the production of pAmpC, ESBLs and carbapenemases have also led to the multidrug-resistance and high potential dissemination of clinical *E*. *cloacae* isolates[23, 24]. Reports on MDR *E*. *cloacae* worsening clinical outcome and prolonging hospitalization duration have been alarmingly increasing since 21^st^ century[7–9]. Our study showed most of the isolates remained susceptible to all tested antibiotics, but ESBL producers hold significantly higher resistance to sulfamethoxazole/trimethoprim and cephalosporins (Table 1). Regarding the clinical characteristics, *E*. *cloacae* bloodstream isolates harboring resistant genes were mostly associated with elderly patients from the surgery or Intensive Care Unit (S1 Table). This also necessitated the cooperative ongoing monitoring on phylogenetic screening and investigation of novel treatments especially for immunocompromised patients to prevent possible expansion of MDR *E*. *cloacae* isolates.

Of all *E*. *cloacae* isolates enrolled, 15.1% (8/53) produced ESBL, which was higher than that in north-eastern USA (10.1%)[3], and pretty higher than that reported by a surveillance conducted in Europe and the USA from 1999 to 2004 with ~5% ESBL-producing *Enterobacter spp*. collected from all sites[12]. Unlike CTX-M-producers most frequent in Latin American countries[13], SHV was the dominant ESBL type with all identical to type 12 in this study (Table 2). The proportion of SHV (75.0%) was higher than that in Guangdong China (58%) [23], and similar to that in Tunisia (77.8%) [24]. SHV-12, one of the most prevalent SHV types in Asia, can confer resistance against β-lactams, fluoroquinolones, aminoglycosides and sulfonamides, and its location and transmission efficiency were correlated closely with the antibiotic resistance of *E*. *cloacae* [23]. What’s worse, the coexistence of carbapenemase and/or pAmpC were detected among over half of the SHV-12-producers (4/6) in our study (Table 3), warranting long-term monitoring on phylogenetic screening and prudent use of antibiotics. Additionally, several reports have found some spreading subclones (ST66, ST78 and ST114) especially ST114, were specifically associated with CTX-M production [25, 26], while in our study only ST78 and ST114 were identified, and both susceptible to all tested antibiotics (Table 3). Besides, distributions of these resistant genes were different in Hospital A and B (Table 2), which suggested that different resistance genes may exhibit geographic difference among *E*. *cloacae* isolates due to various modes of transmission.

PAmpC can preferentially hydrolyze all β-lactams except fourth-generation cephalosporins and carbapenems and may serve as the reservoir for the emergence of antibiotic resistance [27]. The high proportion of MIR/ACT (83.3%) in pAmpC producers (Table 2) was not surprising as it has been reported as the most predominant AmpC gene in the Asia-Pacific area (77.8%)[28]. It is noteworthy the simultaneous production of pAmpC and other β-lactamases in four isolates (ST177, ST191, ST418 and ST528) (as observed in Table 3 with decreased susceptibility to cefepime) could cause quick acquisition and potential spread of transferable resistance determinants and thus complicate the therapeutic treatment for the infections. Factually, the resistance to cefepime could be associated with the presence of SHV-12 or CTX-65, despite in addition to pAmpC, other mechanisms such as TEM-1 alteration or overexpression and/or porin loss could be involved [2, 29], and further molecular studies were demanded. The co-carriage of ESBL, carbapenemase and pAmpC in *E*. *cloacae* was once reported in Taiwan[30], but firstly in Shanghai.

Carbapenemase spread has been increasingly reported worldwide over the last decade[31]. We found one carbapenemase producer (ST418, NDM-1) carried TEM-1 and SHV-12 as well, and the other two (ST191 and ST528, IMP-26) co-carried TEM-1, SHV-12 and DHA-1 (Table 3). Australia has observed an increasing incidence of CRE with the majority being IMP producers[14], which agreed with current study, but the prevailing subtypes were not the same in different regions[14, 30]. IMP-26 was first reported in a *Pseudomonas aeruginosa* isolate in Singapore [32] and few reports appeared later about IMP-26-producing isolates especially *E*. *cloacae* isolates, but interestingly, the two IMP-producers in our study were both detected as IMP-26. It was still noted that IMP-producing *E*. *cloacae* had caused several outbreaks or possible spread in other countries [14, 33, 34], so we should keep continuously monitoring of carbapenems resistance and carbapenemase productions among *E*. *cloacae*. NDM-1 was the dominant carbapenemase of carbapenems-resistant *E*. *cloacae* in Henan China, and in contrast to VIM-1 the most prevalent in Spain and other southern Europe [35, 36]; while only one NDM-1-producer and none VIM-1-producer were found in current study.

A clonality study of *E*. *cloacae* has once denoted the association of distinct clonal groups with genetic lineages of higher prevalence and/or wider geographical spread [25]. However, our study has only demonstrated local *E*. *cloacae* bloodstream isolates did not evolve from a unique ancestral background with 51 STs distinguished from 53 isolates and 8 isolates (15.1%) assigned to 3 CCs (Fig 1). STs harboring two or three types of enzymes shared no alleles in common with each other (Table 3, Fig 1). This implied no clonal dissemination in the region.

There may be one limitation in this study: many hospitals do not preserve clinical isolates regularly in China, so our conclusion based on two hospitals may not be generalizable enough or extrapolated directly to the whole region. However, it provides the step stone for future national researches associated with cooperative surveillance on resistance and resistant mechanisms to control potential further dissemination.

## Conclusions

Our study described the phenotypic and molecular properties of *E*. *cloacae* bloodstream isolates in Shanghai for the first time. Based on the few isolates analyzed, it was noted that antimicrobial resistance was low among them. The simultaneous presence of ESBL, carbapenemase and pAmpC genes detected in 2 *E*. *cloacae* bloodstream isolates was first reported in Shanghai. Genetic diversity revealed no evidence suggesting a spread of these isolates. Active long-term surveillance should be continuously implemented on antimicrobial resistance and consistent prevention and control of *E*. *cloacae*.

## Supporting information

S1 Table Clinical data, rates of drug resistance and molecular characteristics of 53 *E*. *cloacae* from bloodstream infections. (XLSX)
